# Genetic Analysis of Biopsy Tissues from Colorectal Tumors in Patients with Ulcerative Colitis

**DOI:** 10.3390/cancers16193271

**Published:** 2024-09-26

**Authors:** Noriko Yamamoto, Yuji Urabe, Hikaru Nakahara, Takeo Nakamura, Daisuke Shimizu, Hirona Konishi, Kazuki Ishibashi, Misa Ariyoshi, Ryo Miyamoto, Junichi Mizuno, Takeshi Takasago, Akira Ishikawa, Akiyoshi Tsuboi, Hidenori Tanaka, Ken Yamashita, Yuichi Hiyama, Yoshihiro Kishida, Hidehiko Takigawa, Toshio Kuwai, Koji Arihiro, Fumio Shimamoto, Shiro Oka

**Affiliations:** 1 Department of Gastroenterology, Hiroshima University Hospital, Hiroshima 734-8551, Japan; d211116@hiroshima-u.ac.jp (N.Y.); takeo-nakamura@hiroshima-u.ac.jp (T.N.); dshimizu@hiroshima-u.ac.jp (D.S.); hironak@hiroshima-u.ac.jp (H.K.); kishibashi@hiroshima-u.ac.jp (K.I.); misa4235@hiroshima-u.ac.jp (M.A.); ryo4book@hiroshima-u.ac.jp (R.M.); d216467@hiroshima-u.ac.jp (J.M.); takasago@hiroshima-u.ac.jp (T.T.); atsuboi@hiroshima-u.ac.jp (A.T.); hitanaka@hiroshima-u.ac.jp (H.T.); kenyama5@hiroshima-u.ac.jp (K.Y.); kishida1@hiroshima-u.ac.jp (Y.K.); hidehiko@hiroshima-u.ac.jp (H.T.); kuwai@hiroshima-u.ac.jp (T.K.); oka4683@hiroshima-u.ac.jp (S.O.); 2 Department of Clinical and Molecular Genetics, Hiroshima University Hospital, Hiroshima 734-8551, Japan; hnkhr@hiroshima-u.ac.jp; 3 Department of Molecular Pathology, Graduate School of Biomedical and Health Sciences, Hiroshima University, Hiroshima 734-8553, Japan; a-ishikawa@hiroshima-u.ac.jp; 4 Clinical Research Center in Hiroshima, Hiroshima University Hospital, Hiroshima 734-8551, Japan; yhiyama@hiroshima-u.ac.jp; 5 Gastrointestinal Endoscopy and Medicine, Hiroshima University Hospital, Hiroshima 734-8551, Japan; 6 Department of Anatomical Pathology, Hiroshima University Hospital, Hiroshima 734-8551, Japan; arihiro@hiroshima-u.ac.jp; 7 Faculty of Health Sciences, Hiroshima Cosmopolitan University, Hiroshima 734-0014, Japan; simamoto@pu-hiroshima.ac.jp

**Keywords:** ulcerative colitis, ulcerative colitis-associated neoplasia, sporadic neoplasia, biopsy, *TP53* mutation

## Abstract

**Simple Summary:**

Colorectal neoplasia developing from ulcerative colitis mucosa (CRNUC) can be divided into ulcerative colitis-associated neoplasia (UCAN) and non-UCAN; however, it is often difficult to distinguish UCAN from non-UCAN during a biopsy diagnosis. We investigated whether a genomic analysis could improve the diagnostic accuracy of UCAN using biopsy specimens. The pathological diagnosis of biopsy specimens using only hematoxylin and eosin (HE) staining had a sensitivity of 33% and an accuracy of 38% for UCAN diagnosis. On the other hand, the combination of the HE pathology and p53 immunohistochemical staining had a sensitivity of 73% and an accuracy of 85% for UCAN diagnosis, while the combination of HE staining and a *TP53* mutation had a sensitivity of 87% and an accuracy of 88% for UCAN diagnosis. An evaluation of *TP53* mutations in biopsy specimens may be useful for diagnosing UCAN. However, further studies with larger sample sizes are required before this can be applied in clinical practice.

**Abstract:**

Background/Objectives: Colorectal neoplasia developing from ulcerative colitis mucosa (CRNUC) can be divided into ulcerative colitis-associated neoplasia (UCAN) and non-UCAN; however, it is often difficult to distinguish UCAN from non-UCAN during a biopsy diagnosis. We investigated whether a genomic analysis could improve the diagnostic accuracy of UCAN using biopsy specimens. Methods: In step 1, 14 CRNUCs were used to examine whether the genomic landscape of biopsy and resection specimens matched. In step 2, we investigated the relationship between the genomic landscapes and the pathological diagnosis of 26 CRNUCs. The cancer genome was analyzed by deep sequencing using a custom panel of 27 genes found to be mutated in our previous CRNUC analysis. Results: In step 1, of the 27 candidate genes, 14 were mutated. The concordance rate of the pathogenic mutations in these 14 genes between the biopsy and resection specimens was 29% (4/14), while that of the pathogenic mutations in *TP53* and *KRAS* was 79% (11/14). In step 2, the pathological diagnosis of biopsy specimens using only hematoxylin and eosin (HE) staining had a sensitivity of 33% and an accuracy of 38% for UCAN diagnosis. On the other hand, the combination of the HE pathology and p53 immunohistochemical staining had a sensitivity of 73% and an accuracy of 85% for UCAN diagnosis, while the combination of HE staining and a *TP53* mutation had a sensitivity of 87% and an accuracy of 88% for UCAN diagnosis. Conclusions: An evaluation of *TP53* mutations in biopsy specimens may be useful for diagnosing UCAN. However, further studies with larger sample sizes are required before this can be applied in clinical practice.

## 1. Introduction

In recent years, the prevalence of ulcerative colitis (UC) has increased worldwide [1,2]; however, recent advances in the treatment of UC have led to an increase in the proportion of patients with UC in remission, reducing the need for a total proctocolectomy for refractory UC [3,4,5]. On the other hand, there has been an increase in the number of surgeries for UC-associated dysplasia (UCAD) or UC-associated carcinoma (UCAC), due to the increase in the avoidance rate of a total proctocolectomy and the prolonged duration of UC disease [5]. In the Asian region, the prevalence of UC-associated cancer in patients with UC has been reported to be 0.85% overall, 0.02% after 10 years of disease duration, 4.81% after 20 years, and 13.91% after 30 years [6]. Several guidelines recommend treatment for UC-associated neoplasia (UCAN); an endoscopically visible low-grade dysplasia (LGD) can be resected endoscopically [7], while a total proctocolectomy is recommended for a high-grade dysplasia (HGD) or UCAC [8,9].

A sporadic neoplasia (SN), which can arise in patients with UC, has a better prognosis compared to UCAN [10,11]; a local endoscopic resection or partial colectomy is recommended, as for colorectal neoplasia developing in patients without UC [12,13,14]. Due to the different treatment recommendations, it is important to distinguish between UCAN and non-UCAN before resection; however, differentiating UCAN from non-UCAN by endoscopic findings and biopsy pathology is often difficult [15]. UCANs have a different carcinogenesis pathway than non-UCANs; UCANs can lead to carcinoma through the inflammation–dysplasia–carcinoma pathway, while non-UCANs can lead to carcinoma through the adenoma–carcinoma pathway or the serrated pathway [16,17]. Therefore, UCANs and non-UCANs have different genetic mutations that occur at an early stage. Our previous study indicated that colorectal neoplasia developing from UC mucosa (CRNUC) can be divided into two groups: neoplasias with *TP53* mutations that develop through the inflammation–dysplasia–carcinoma pathway, and those with *KRAS* mutations that develop through other pathways [18]. Several previous studies have also showed a higher frequency of *TP53* mutations in colitis-associated colorectal carcinoma (CAC) and a higher frequency of *KRAS* mutations in sporadic colorectal carcinoma (CRC) [19].

Therefore, this study aimed to investigate whether the genetic analysis of neoplasia biopsy specimens at the time of endoscopy in patients with UC has an additive effect on the pathological diagnosis of UCAN or non-UCAN before resection.

## 2. Materials and Methods

### 2.1. Patients and Sample Preparation

**Figure 1:**
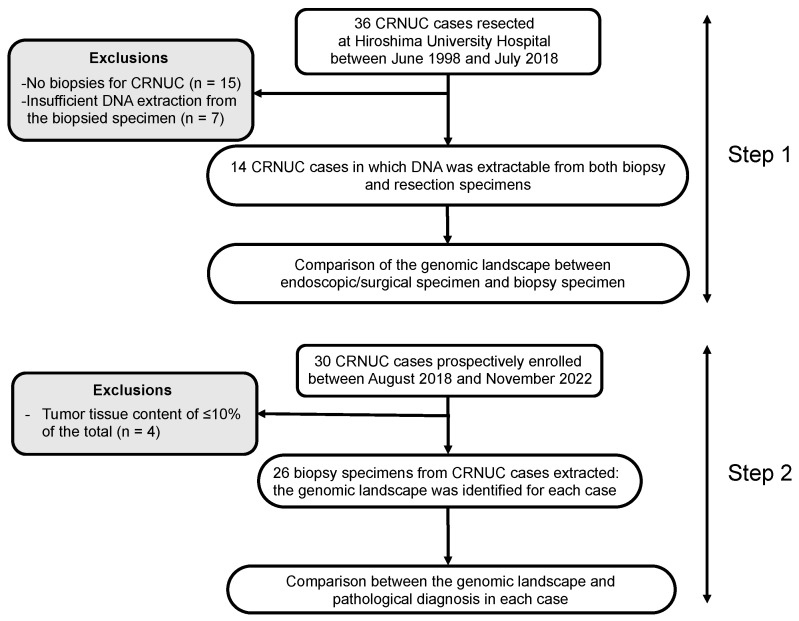
Flow chart of enrolled lesions. In step 1, preoperative biopsy specimens were used from resected specimens of colorectal neoplasia developing from ulcerative colitis of mucosa (CRNUC) that were previously identified through endoscopic or surgical resection, and whose genomic landscape was determined in earlier research. In step 2, preoperative biopsy specimens, different from those used in step 1 and obtained through endoscopic or surgical resection, were utilized as independent samples.

A flow chart of this study’s design is shown in Figure 1. Of the 36 early-stage CRNUCs (HGD and T1 carcinoma) resected at Hiroshima University Hospital between June 1998 and July 2018, for which genetic landscapes in the resected specimens had been examined in our previous study, 14 CRNUCs were included in step 1 of this study. The remaining 22 CRNUCs were not biopsied and were subsequently excluded from this study. In the 14 included CRNUCs, we determined if the genetic landscapes of the biopsy specimens matched the genetic landscapes of the resected specimens.

In step 2 of this study, of the 30 CRNUCs that were biopsied and afterward resected at Hiroshima University Hospital between August 2018 and November 2022, 26 CRNUCs were included; 4 CRNUCs were excluded due to tumor tissue in <10% of the biopsy specimens. These 26 CRNUCs were used to compare the pathological diagnosis, p53 immunostaining status, and genetic landscapes of the biopsy specimens with the final diagnosis of the resected specimens.

### 2.2. Histopathological Assessment

All the resected and biopsied specimens were fixed in 10% formalin, sliced into 2 mm sections, embedded in paraffin, serially sectioned, stained with hematoxylin and eosin (HE), and examined microscopically. In all the cases, the histopathological diagnosis was confirmed by two or more gastrointestinal pathologists. The pathological diagnosis of UCANs using HE staining included the pathological features indicated in the recent European Crohn’s and Colitis Organisation histopathological statements: associated flat dysplasia (no sharp delineation), irregular neoplastic glands (varying configuration, size, and diameter) with varying amounts of stroma, increased (mononuclear) lamina propria inflammation, and a mixture of benign/dysplastic crypts at the surface [20]. A histopathological diagnosis of UCAN was made when a specimen had at least one of these features, while the tumor biopsy specimens with none of these features were diagnosed as non-UCANs.

In the evaluation of the p53 immunohistochemical staining (IHC) of the biopsy specimens, the cases strongly positive for p53 were defined as UCANs. Furthermore, the cases with pathogenic *TP53* mutations were defined as UCANs. In evaluating the combination of HE staining and p53 IHC or *TP53* mutations, the diagnosis made by HE staining took precedence over the p53 IHC or *TP53* mutation status. The diagnosis of UCAN for the combination of HE staining and p53 IHC or *TP53* mutations was defined as the cases with strongly positive p53 IHC or pathogenic *TP53* mutations, if the diagnosis of UCAN could not be made by HE staining of the biopsy specimen.

The histopathological diagnosis of the resected specimens for UCAN was made using Ki67 and p53 IHC in addition to the pathological features with HE staining. In UCANs, Ki67-positive cells are mainly found at the basal side of the mucosa, and tumor cells differentiate towards the superficial side of the mucosa, which is known as the “bottom-up morphology”. Conversely, in SNs, particularly in conventional tubular adenomas, Ki67-positive cells are mainly found at the superficial zone of the mucosal layer, and tumor cells differentiate towards the basal side of the mucosa, which is known as the “top-down morphology”. Additionally, UCANs show a significantly higher degree of p53 expression than sporadic adenomas [21], which is also useful for differentiating between UCANs and SNs. We defined the pathological diagnosis with p53 and Ki67 IHC of the resection specimen as the final diagnosis. The CRNUCs that were difficult to distinguish as either UCANs or SNs by the resection specimens, even by multiple gastrointestinal pathologists, were excluded from this study.

### 2.3. Tissue Collection and DNA Extraction

We used several 10 μm thick slides prepared from formalin-fixed paraffin-embedded (FFPE) biopsied tissues from the tumor and the surrounding non-tumor area. We used a GeneRead DNA FFPE Kit (Qiagen, Valencia, CA, USA) to extract DNA from these tissues, and a Qubit 1.0 Fluorometer (Life Technologies, Grand Island, NY, USA) to determine the DNA concentrations. In addition, we determined the quantity and quality of the FFPE-derived DNA samples by calculating the normalized DNA integrity scores (ΔΔCq) via a quantitative polymerase chain reaction analysis using an Agilent NGS FFPE QC Kit (Agilent Technologies, Santa Clara, CA, USA).

### 2.4. Target Enrichment and Next-Generation Sequencing

**Figure 2:**
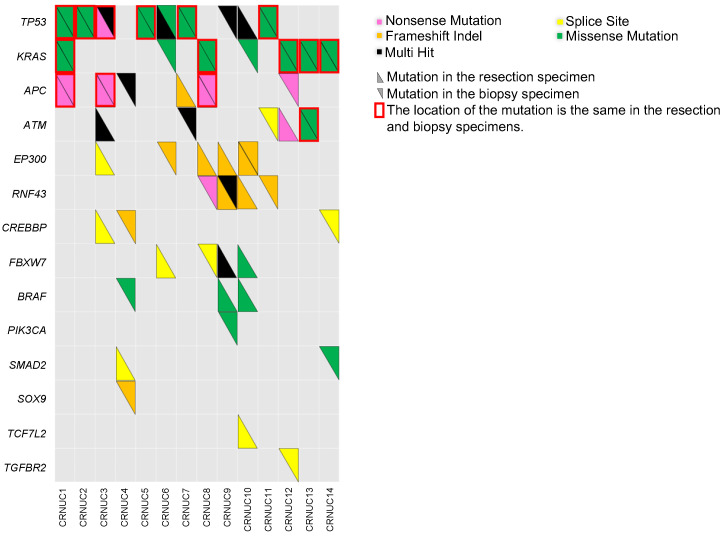
The genomic landscapes of the resected and biopsy specimens of the 14 CRNUC cases in step 1. The panel contains the mutation patterns of 14 genes from the 14 CRNUCs in step 1. The pink, yellow, orange, green, and black cells indicate nonsense mutations, splice site mutations, frameshift insertions/deletions, missense mutations, and multiple hits, respectively. The upward triangles represent mutations in the resection specimens. The downward triangles represent mutations in the biopsy specimens. The cells framed by red rectangles indicate that the mutation sites match in the resected and biopsy specimens. In total, 29% (4/14) of the CRNUC cases had the same mutation status for all the detected genes in the resected and biopsy specimens. Furthermore, 79% (11/14) of the CRNUC cases had the same mutation status for *TP53* and *KRAS* in the resected and biopsy specimens. CRNUC, colorectal neoplasia developing from ulcerative colitis mucosa.

We developed a sequencing library based on DNA extracted from the tumor and non-tumor mucosa using a SureSelect XT HS Kit (Agilent Technologies, Santa Clara, CA, USA) after fragmenting the DNA into 150–200 base pairs using an XT Low Input Enzymatic Fragmentation Kit (Agilent Technologies, Santa Clara, CA, USA). The amount of DNA was measured using a TapeStation D1000 (Agilent Technologies, Santa Clara, CA, USA) before hybridization, which was used if the prepared library was >500 ng. DNA was not obtained from 11 samples. To perform target capture, we used the SureSelect XT Target Enrichment System (Agilent Technologies, Santa Clara, CA, USA) for 27 genes with mutations detected by Matsumoto et al. [18] (Table S1). The resulting pooled libraries were sequenced by paired-end reads using the HiSeq X platform (Illumina, San Diego, CA, USA), following a quality control check with a High Sensitivity D1000 ScreenTape Assay (Agilent Technologies, Santa Clara, CA, USA) (Figure 2).

### 2.5. Variant Detection

Sequencing reads were pre-processed using fastp v0.20 and mapped to hg19 using BWA-MEM v0.7.17 [22]. GATK best practice was used for variant calling. To reduce false positives, somatic mutations were defined as read depths >50 and variant allele frequencies >4%. Copy number analysis was performed using CNVkit v0.9.9 and PureCN v2.0.1 [23]. Vcf2maf v1.6.21 (https://zenodo.org/record/1185418#.Y_W6cC_3IUs (accessed on 26 April 2024)), oncokb-annotator v3.2.1 (https://github.com/oncokb/oncokb-annotator/releases (accessed on 26 April 2024)), and InterVar v2.2.2 [24] were used for annotation. We defined alterations as mutations, amplifications, or deletions that were classified as oncogenic or likely oncogenic status in OncoKB (https://www.oncokb.org (accessed on 26 April 2024)), or pathogenic or likely pathogenic status in ClinVar (https://www.ncbi.nlm.nih.gov/clinvar/ (accessed on 26 April 2024)). R package maftools v2.8.5 (https://bioconductor.org/packages/release/bioc/html/maftools.html (accessed on 26 April 2024)) was used for plotting.

### 2.6. Methods for Assessing the Diagnosis of UCAN by p53 IHC, TP53 Genetic Analysis, and HE Staining

For the evaluation of the HE staining of the biopsy specimens, the “sensitivity” of the diagnosis of UCAN was defined as the ratio of the number of cases correctly diagnosed as UCAN by HE staining alone to the number of cases that were UCAN. For the evaluation of the p53 staining of the biopsy specimens, UCAN was assumed for the cases that could be diagnosed as UCAN by the HE staining of the biopsy specimens alone, or the cases that were not diagnosed as UCAN by HE staining but were strongly positive in the p53 IHC. For the evaluation of a *TP53* mutation in the biopsy specimens, the cases that could be diagnosed as UCAN by HE staining of biopsy specimens alone, or the cases that could not be diagnosed as UCAN by HE staining but were positive for a *TP53* mutation were assumed to be UCAN.

### 2.7. Ethics Statements

This study was performed in accordance with the principles of the Declaration of Helsinki, and this study’s protocol was approved by the Institutional Review Board of Hiroshima University (approval number: E2022-0317; registration date: 12 May 2023).

### 2.8. Statical Analysis

The categorical variables were expressed as frequencies and proportions and were compared using the chi-squared and Fisher’s exact tests. The kappa value was evaluated based on the strength of agreement reported by Landis et al. [25]. The statistical analyses were performed using JMP version 17 (SAS Institute Inc., Cary, NC, USA); *p* < 0.05 was considered statistically significant.

## 3. Results

### 3.1. Concordance of Mutations in Biopsy and Resected Specimens (Step 1)

The clinicopathological features of the CRNUCs in step 1 are shown in Table S2. We compared the genomic landscape of the biopsy specimens with that of the resected specimens in the same cases. Figure 2 shows the genomic landscapes of the biopsy and resected specimens of the 14 CRNUCs in step 1. Of the 27 candidate genes, 14 genes were found to be mutated. The concordance rate of the pathogenic mutations in these 14 genes between the biopsy and resected specimens was 29% (4/14), while that of the pathogenic mutations in *TP53* and *KRAS* was 79% (11/14) (Figure 2).

### 3.2. Genomic Landscapes of Somatic Mutations in Biopsy Specimens (Step 2)

**Figure 3:**
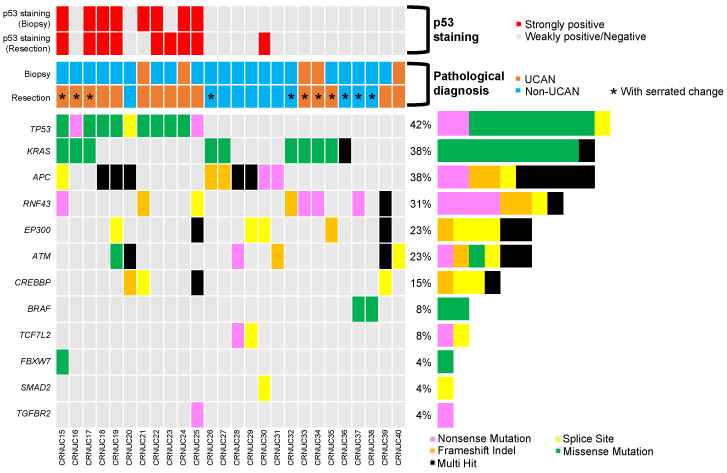
The pathological diagnosis and genomic landscapes of the 26 CRNUC cases in Step 2. (Upper panel) The p53 staining status of the biopsy or resected specimens. The red and gray cells indicate strongly positive and weakly positive/negative p53 staining status, respectively. (Middle panel) The pathological diagnosis of the biopsy and resected specimens. The pathological diagnosis of the biopsy specimens in this figure refers to the pathological diagnosis by hematoxylin and eosin (HE) staining only, and the pathological diagnosis of the resection specimens refers to the pathological diagnosis by HE staining with a p53 and Ki67 immunostaining assessment. The brown and light-blue cells indicate a UCAN and non-UCAN diagnosis, respectively. The asterisks in the cells indicate CRNUCs with serrated changes. (Lower panel) The mutation patterns of 12 genes from the 26 CRNUCs in step 2. The pink, yellow, orange, green, and black cells indicate nonsense mutations, splice site mutations, frameshift insertions/deletions, missense mutations, and multiple hits, respectively. The bar graph on the right shows the frequency of mutations in each gene. CRNUC, colorectal neoplasia developing from ulcerative colitis mucosa; UCAN, ulcerative colitis-associated neoplasia.

The clinicopathological features of the CRNUCs in step 2 are shown in Table S3. As for the genomic landscapes of the biopsy specimens, the most common mutated gene was *TP53*, which was found to be mutated in 42% (11/26) of the cases (Figure 3). Other mutated genes found in >10% of the cases were *KRAS*, *APC*, *RNF43*, *EP300*, *ATM*, and *CREBBP*, which were found to be mutated in 38% (10/26), 38% (10/26), 31% (8/26), 23% (6/26), 23% (6/26), and 15% (4/26) of the cases. *TP53* mutations were seen in 67% (10/15) of UCANs and 9% (1/11) of non-UCANs, while *KRAS* mutations were seen in 40% (6/15) of UCANs and 36% (4/11) of non-UCANs. The *TP53* and *KRAS* genes were both mutated in three CRNUCs, all of which were UCANs.

### 3.3. Accuracy of the Pathological Diagnosis and TP53 Mutation of Biopsy Specimens for Differentiation between UCAN and Non-UCAN

The biopsy specimen pathology with HE staining alone had a sensitivity of 33% for the diagnosis of UCAN, and an accuracy of 38% for differentiating UCAN from non-UCAN (Table S4). The accuracy of diagnosing UCAN/non-UCAN from the biopsy specimens with HE staining alone was 38% (10/26) (Table S4), while that by p53 IHC and *TP53* mutation was 73% (19/26, *p* = 0.026) (Table S5) and 77% (20/26, *p* = 0.012), respectively (Table S6). The combination of the HE pathology with other methods improved the UCAN diagnosis; combining the HE pathology with p53 IHC in the biopsy specimens achieved a sensitivity of 73%, an accuracy of 85% (22/26), and a kappa value of 0.6994, while combining the HE pathology with *TP53* mutation analysis resulted in a sensitivity of 87%, an accuracy of 88% (23/26), and a kappa value of 0.7665.

### 3.4. Correlation between KRAS/BRAF Mutations of Biopsy Specimens and Features of the CRNUCs

We also examined the correlation between the clinicopathological features and *KRAS/BRAF* mutations in the CRNUCs. *KRAS* mutations were found in 38% (10/26) of the CRNUCs, while *BRAF* mutations were found in 8% (2/26). There was no association between the presence of *KRAS/BRAF* mutations and the duration of UC disease, either in the UCANs or non-UCANs (Figure S1). Regarding the clinical features of early CRNUCs (LGD, HGD, or T1 carcinoma), there was no significant difference in the tumor location, inflammation of background mucosa, clinical course of UC, or type of UC between the patients with and without *KRAS/BRAF* mutations (Figure S2). Regarding the endoscopic findings of the early CRNUCs, the CRNUCs with *KRAS/BRAF* mutations tended to be less flat in their morphology, while there was no significant difference in tumor color between the patients with and without *KRAS/BRAF* mutations (Figure S3). Focusing on the presence or absence of serrated changes in the resected specimens, we found that the CRNUCs with *KRAS/BRAF* mutations tended to have serrated changes. When regarding the *KRAS/BRAF* mutations in the biopsy specimen as a predictive marker for histological serrated changes in the resected specimen, the sensitivity, accuracy, and kappa value were 94%, 88%, and 0.75, respectively (Table 1).

## 4. Discussion

This study reveals that the genomic landscape of CRNUC biopsy specimens and post-endoscopic submucosal dissection specimens does not match for all genes; however, the mutations in *KRAS* and *TP53* are almost identical. Additionally, the diagnostic accuracy for UCAN using biopsy specimens with HE staining alone is low, but improves with the addition of p53 staining and *TP53* mutation analysis. Furthermore, it is found that the accuracy of p53 staining and *TP53* mutation analysis for diagnosing UCAN is almost equivalent. Although *KRAS* mutations are not useful for the diagnosis of UCAN, they are correlated with the presence or absence of serration in the lesions.

With respect to UCANs, HGDs and carcinomas are, in principle, indications for a total proctocolectomy [8,9]. On the other hand, for SNs, local resection (endoscopic or surgical) is generally indicated, even for HGDs or carcinomas [12,13,14]. If we could distinguish between UCANs and SNs based on the endoscopic findings and biopsy results alone, it could help select treatment options for CRNUCs. Therefore, improving the diagnostic value of biopsy specimens is important.

The histological diagnoses of biopsy specimens and resected specimens can differ because biopsy specimens are taken from only a part of the tumor. The concordance rate of the pathological diagnosis between biopsy and resected specimens in colorectal tumors has been reported to be 43–88% [26,27,28]. On the other hand, Isaka et al. investigated the concordance rate of the mutations detected by amplicon-based massively parallel sequencing between a transbronchial biopsy and surgically resected specimens in fresh-frozen primary tumor specimens of non-small-cell lung cancer [29]. Moreover, in normal-type colon cancer, the concordance rates for *TP53* and *KRAS* mutations have been reported to be 100% [30,31] and 97–100% [30,32,33]. However, there are no reports examining the concordance rate of mutations between endoscopic biopsy specimens and surgically/endoscopically resected specimens in FFPE sections of CRNUC. Therefore, in this study, we investigated the concordant mutations identified by deep sequencing in biopsy specimens and endoscopically/surgically resected specimens. Our results show the concordance rates for *KRAS* and *TP53* mutations between the biopsy specimens and endoscopically/surgically resected specimens were high. This could be attributed to the fact that *KRAS* and *TP53* mutations are present from the early stages of tumor clonal evolution, making these mutations detectable in almost all tumor cells. Therefore, *KRAS* and *TP53* mutations in biopsy specimens are considered to reflect the genomic mutations of the entire tumor.

The diagnostic accuracy of UCAN with HE staining alone has been reported to be low. Therefore, several studies have reported methods to improve the accuracy of biopsy diagnosis in CRNUC [34,35,36]. Since *TP53* mutations are considered to play a role in inflammation-associated carcinogenesis, the use of p53 IHC to enhance diagnostic accuracy has been reported. The frequency of p53 expression in UCAN has been reported to be 0–31%, 59–80%, and 57–91% for LGD, HGD, and CAC, respectively, and the frequency of p53 expression in non-UCAN to be 4–22% and 55–61% for adenoma and CRC, respectively; the frequency of p53 expression in UCAN is known to be higher than in non-UCAN [37,38,39,40]. Therefore, we assumed that strongly positive p53 biopsy specimens were UCAN in our evaluation. Previous studies have reported that the frequency of *TP53* mutations in colitis-associated neoplasia were 33%, 63%, and 80–89% in LGD, HGD, and CAC, respectively [41,42,43,44], and that the *TP53* mutation frequency in non-UCAN was 52–61% [41,42,43], even in carcinoma. Moreover, in our study, the diagnostic accuracy of p53 IHC for UCAN/non-UCAN in the biopsy specimens was 73%, and the diagnostic accuracy using both HE staining and p53 IHC was 85%. Thus, p53 IHC is a useful method for the biopsy diagnosis of UCAN; however, there is a need to further improve the diagnostic accuracy of UCAN. In our previous study, our genomic analysis of endoscopic and surgical resection specimens of CRNUC indicated that *TP53* mutations and *KRAS* mutations are mutually exclusive, suggesting that *TP53* mutations may serve as a marker for UCAN. Therefore, assuming that *TP53* mutations serve as a marker for the diagnosis of UCAN, we evaluated the diagnosis of UCAN/non-UCAN using biopsy specimens. In this study, the diagnostic accuracy was 77% with *TP53* mutations alone, and 88% when combining the HE pathology with *TP53* mutation analysis. These results suggest that the diagnostic accuracy of UCAN/non-UCAN in biopsy specimens is comparable between the p53 IHC and *TP53* mutation analysis. In this study, 5 of the 15 UCAN cases were negative for *TP53* mutations. One reason for the negative *TP53* mutations could be intra-tumor heterogeneity, as sporadic colorectal tumors are known to have intra-tumor heterogeneity [27,45], as does UCAN [46]. Another possible reason could be that the cases without *TP53* mutations may have followed an oncogenic pathway that was not mediated by *TP53* mutations. According to previous reports analyzing the genetic mutations in resected specimens of colitis-associated neoplasia, the existence of oncogenic pathways that are not mediated by *TP53* mutations has been noted [47,48]. Previous reports have shown that *IDH1* and *MYC* mutations are common in UCAN [47,48]. Although it is possible that the *TP53* mutation-negative UCAN cases in this study also had the mutations mentioned above, we did not search for *IDH1* or *MYC* mutations in the present study.

The frequency of *KRAS* mutations in the UCAN and non-UCAN cases in this study were similar, which differs from the results of previous studies [18,19]. This result was surprising; therefore, we examined the histological characteristics of the cases with *KRAS* mutations. As a result, the CRNUCs with *KRAS* mutations tended to have serrated histological changes, which were also observed in the CRNUCs with *BRAF* mutations. Furthermore, *KRAS* and *BRAF* mutations were mutually exclusive. Previous studies of serrated epithelial changes (SECs) in UC have reported that SECs are related to long-standing UC, and UC patients with SECs are more likely to develop UCAN than UC patients without SECs [49,50]. Furthermore, Singhi et al. reported that patients with SECs in UC had *TP53* mutations more frequently than sporadic serrated polyps, indicating the possibility of the SECs being the precursor of UCAN [51]. In addition, the previously reported *KRAS* mutation frequency in SECs was 39–50%, and the *BRAF* mutation frequency was 9–15% [52,53]. In the present study, 60% (6/10) of the *KRAS* mutation-positive lesions were UCANs, and all of these had serrated changes, suggesting that SECs may be a precursor of these UCANs. In this study, two CRNUCs were *BRAF* mutation positive, and both were diagnosed with sporadic serrated neoplasia. In the future, it will be necessary to investigate the mechanisms by which serrated changes occur in UCAN and the involvement of *KRAS/BRAF* mutations in this process.

This study has some limitations. First, this is a single-centered retrospective study, and the number of cases is limited. Second, the biopsy and resection specimens used in this study are FFPE specimens, and the quality of the DNA samples is low. Third, the concordance rate between the biopsy and resection specimens for all pathogenic mutations is 29%, and even for TP53 and KRAS it is 79%, indicating that biopsy specimens may not fully reflect all the pathogenic mutations present in resection specimens. The heterogeneity of tumors and the potential for sampling errors may result in misdiagnosis through biopsy, and could also be a contributing factor to the low concordance rate between the biopsy and resection specimens described above.

## 5. Conclusions

In conclusion, the evaluation of *TP53* mutations in biopsy specimens may be useful for diagnosing UCAN. The results of this study can improve the diagnostic accuracy using biopsy specimens, help avoid unnecessary invasive treatments, reduce medical costs, and prevent complications associated with treatment. However, as highlighted in previous reports, tumor heterogeneity and the possibility of sampling errors in tumor biopsies remain significant challenges [27,45,46]. These factors likely contributed to the discrepancies in the genomic mutations observed between the biopsy and resection specimens in our study. Given the insufficient concordance rate of the genomic mutations between the biopsy and resection specimens, the current sample size may be inadequate for making critical decisions about highly invasive treatments, such as a total colectomy. Therefore, to ensure the practical application of these findings in clinical settings, larger-scale studies with expanded sample sizes are needed. Such studies would help to establish clearer guidelines on how genomic analysis results should be incorporated into diagnostic criteria.

## Figures and Tables

**Table 1:** Correlation between histological serrated changes in resection specimens and *KRAS/BRAF* mutations of biopsy specimens in 40 CRNUCs in step 1 and step 2.

*KRAS/BRAF* Mutation	Serrated Change (%)		Total (%)	
	+	−		
**+/−**	13 (76)	4 (24)	17 (100)	*p* < 0.01 ^ab^
**−/+**	3 (100)	0 (0)	3 (100)	
**−/−**	1 (5)	19 (95)	20 (100)	

^a^ Categorical variables were compared using Fisher’s exact tests. ^b^ We compared a group that was positive for either a *KRAS/BRAF* mutation with a group that was negative for both *KRAF/BRAF* mutations. CRNUC, colorectal neoplasia developing from ulcerative colitis mucosa.

## Data Availability

The data that support the findings of this study are not openly available due to the privacy of patients and are available from the corresponding author upon reasonable request. The data are in controlled access data storage at Hiroshima University Hospital.

## Supplementary Materials

The following supporting information can be downloaded at: https://www.mdpi.com/article/10.3390/cancers16193271/s1, Figure S1: Correlation between *KRAS/BRAF* mutation status of biopsy specimens and UC duration in 45 CRNUC cases in step 1 and step 2; Figure S2: Correlation between *KRAS/BRAF* mutation status in biopsy specimens and clinical features of 35 early CRNUCs; Figure S3: Correlation between *KRAS/BRAF* mutation status in biopsy specimens and endoscopic findings of 40 early CRNUCs; Table S1: Details of 40 genes included in multi-gene panel in this study; Table S2: Clinicopathological features of colorectal neoplasia in step 1 (n = 14); Table S3: Clinicopathological features of colorectal neoplasia in step 2 (n = 26); Table S4: Correlation between pathological diagnosis of resected specimens and pathological diagnosis of biopsy specimens in 26 CRNUCs from step 2; Table S5: Correlation between pathological diagnosis of resected specimens and p53 staining of biopsy specimens in 26 CRNUCs in step 2; Table S6: Correlation between pathological diagnosis of resected specimens and *TP53* mutation status of biopsy specimens from 26 CRNUCs in step 2.
