# Supplementary material for: Genetic Analysis of Biopsy Tissues from Colorectal Tumors in Patients with Ulcerative Colitis
Source: Cancers (Basel). 2024 Sep 26;16(19):3271. doi: 10.3390/cancers16193271 (PMC11475702; doi:10.3390/cancers16193271)
Supplement: Supplementary file 1 [file cancers-16-03271-s001.zip › cancers-3210575-supplementary.pdf]

Supplementary Materials

Genetic Analysis of Biopsy Tissues from Colorectal Tumors in Patients with Ulcerative Colitis

Noriko Yamamoto [1], Yuji Urabe [1,*], Hikaru Nakahara [2], Takeo Nakamura [1], Daisuke Shimizu [1], Hirona Konishi [1], Kazuki Ishibashi [1], Misa Ariyoshi [1], Ryo Miyamoto [1], Junichi Mizuno [1], Takeshi Takasago [1], Akira Ishikawa [3], Akiyoshi Tsuboi [1], Hidenori Tanaka [1], Ken Yamashita [1], Yuichi Hiyama [4], Yoshihiro Kishida [1], Hidehiko Takigawa [1], Toshio Kuwai [1,5], Koji Arihiro [6], Fumio Shimamoto [7] and Shiro Oka [1]

[1] Department of Gastroenterology, Hiroshima University Hospital, Hiroshima 734-8551, Japan; d211116@hiroshima-u.ac.jp (N.Y.); takeo-nakamura@hiroshima-u.ac.jp (T.N.); dshimizu@hiroshima-u.ac.jp (D.S.); hironak@hiroshima-u.ac.jp (H.K.); kishibashi@hiroshima-u.ac.jp (K.I.); misa4235@hiroshima-u.ac.jp (M.A.); ryo4book@hiroshima-u.ac.jp (R.M.); d216467@hiroshima-u.ac.jp (J.M.); takasago@hiroshima-u.ac.jp (T.T.); atsuboi@hiroshima-u.ac.jp (A.T.); hitanaka@hiroshima-u.ac.jp (H.T.); kenyama5@hiroshima-u.ac.jp (K.Y.); kishida1@hiroshima-u.ac.jp (Y.K.); hidehiko@hiroshima-u.ac.jp (H.T.); kuwai@hiroshima-u.ac.jp (T.K.); oka4683@hiroshima-u.ac.jp (S.O.)

[2] Department of Clinical and Molecular Genetics, Hiroshima University Hospital, Hiroshima 734-8551, Japan; hnkhr@hiroshima-u.ac.jp

[3] Department of Molecular Pathology, Graduate School of Biomedical and Health Sciences, Hiroshima University, Hiroshima 734-8553, Japan; a-ishikawa@hiroshima-u.ac.jp

[4] Clinical Research Center in Hiroshima, Hiroshima University Hospital, Hiroshima 734-8551, Japan; yhiyama@hiroshima-u.ac.jp

[5] Gastrointestinal Endoscopy and Medicine, Hiroshima University Hospital, Hiroshima 734-8551, Japan

[6] Department of Anatomical Pathology, Hiroshima University Hospital, Hiroshima 734-8551, Japan; arihiro@hiroshima-u.ac.jp

[7] Faculty of Health Sciences, Hiroshima Cosmopolitan University, Hiroshima 734-0014, Japan; simamoto@pu-hiroshima.ac.jp

* Correspondence: beyan13@hiroshima-u.ac.jp; Tel.: +81-82-257-5193

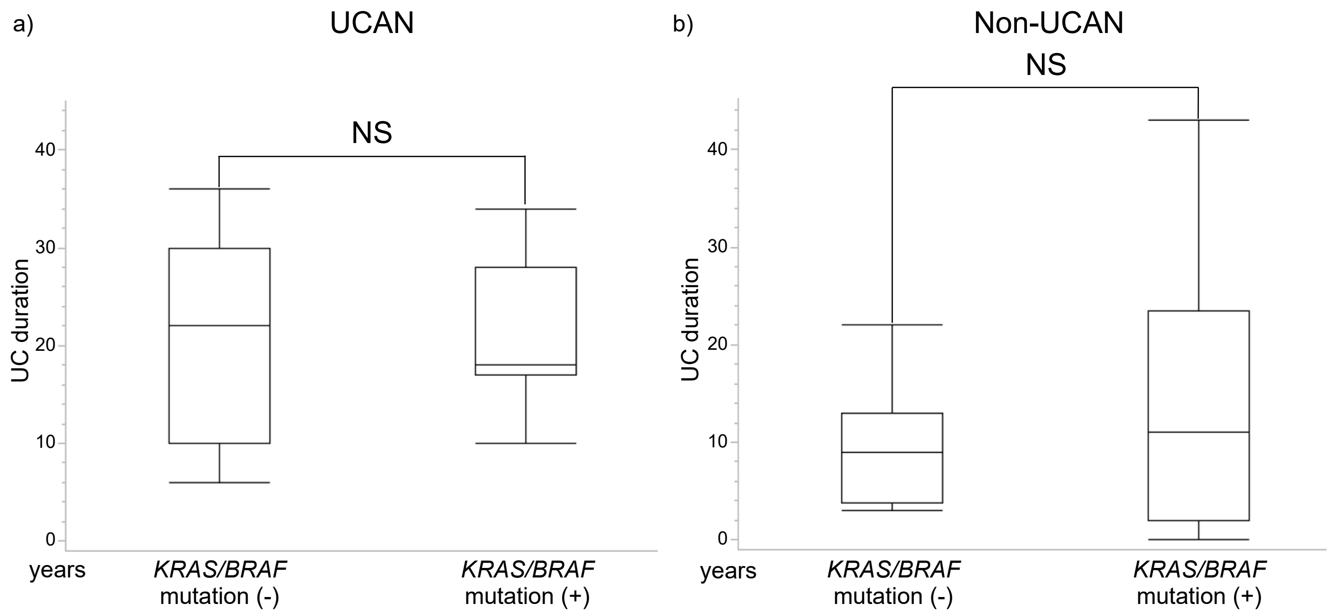

Figure S1: Correlation between *KRAS*/*BRAF* mutation status of biopsy specimens and UC duration in the 45 CRNUC cases in step 1 and step 2. The vertical axis indicates the duration of UC. a) Correlation between *KRAS*/*BRAF* mutation of the biopsy specimens and UC duration in UCANs. b) Correlation between *KRAS*/*BRAF* mutation of the biopsy specimens and UC duration in non-UCANs. CRNUC, colorectal neoplasia developing from ulcerative colitis mucosa; UCAN, ulcerative colitis-associated neoplasia; NS, not significant.

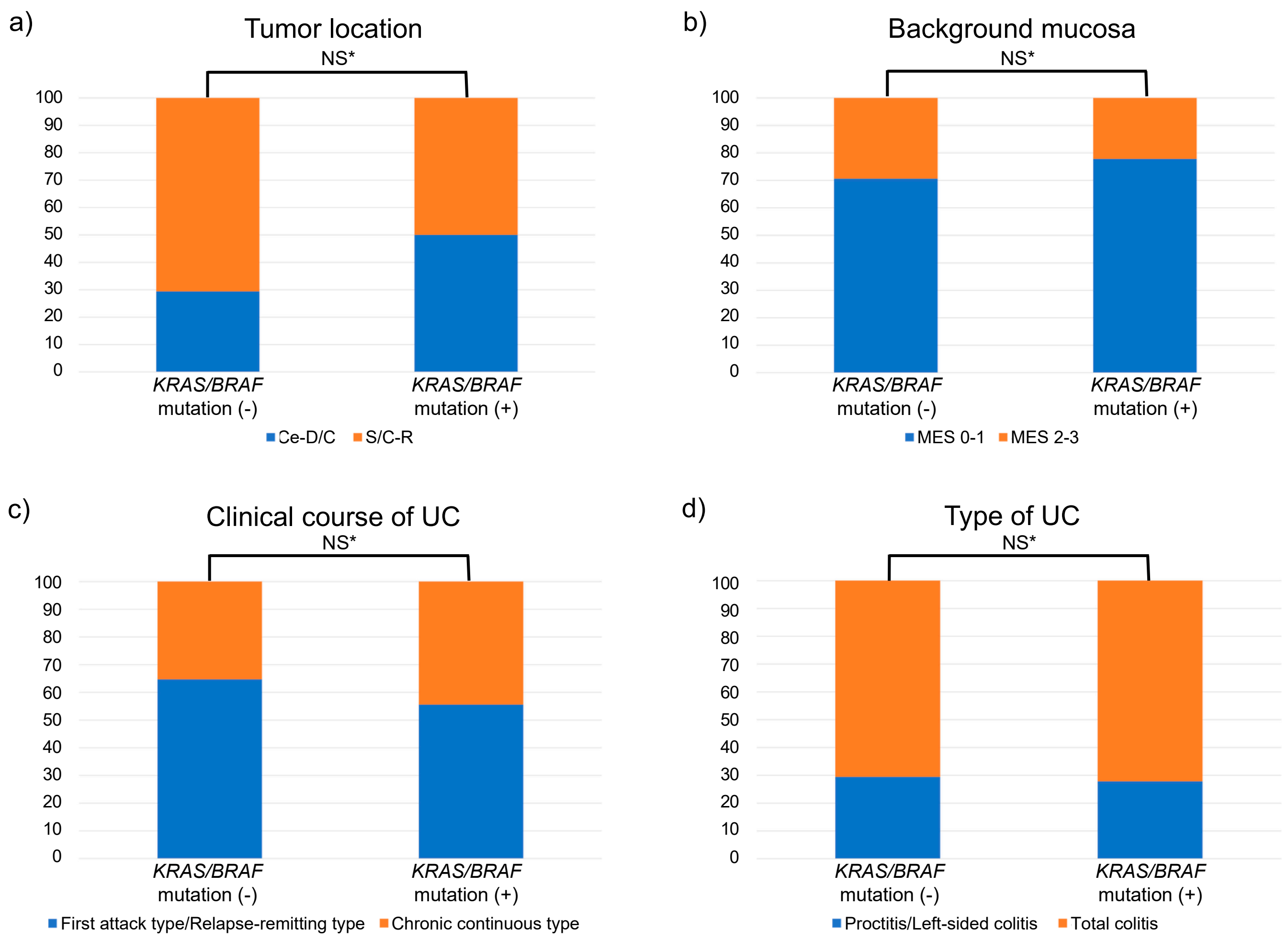

Figure S2: Correlation between *KRAS*/*BRAF* mutation status in biopsy specimens and the clinical features of 35 early CRNUCs. The bar graphs show the correlation between *KRAS*/*BRAF* mutation in biopsy specimens and a) tumor location (Ce–D/C versus [vs.] S/C–R), b) MES of background mucosa (MES0–1 vs. MES2–3), c) clinical course of UC (first attack type vs. chronic continuous type), and d) type of UC (proctitis/left-sided colitis vs. total colitis). CRNUC, colorectal neoplasia developing from ulcerative colitis mucosa; UC, ulcerative colitis; Ce, cecum; D/C, descending colon; S/C, sigmoid colon; R, rectum; MES, Mayo endoscopic subscore; NS, not significant. *Categorical variables were compared using Fisher's exact tests.

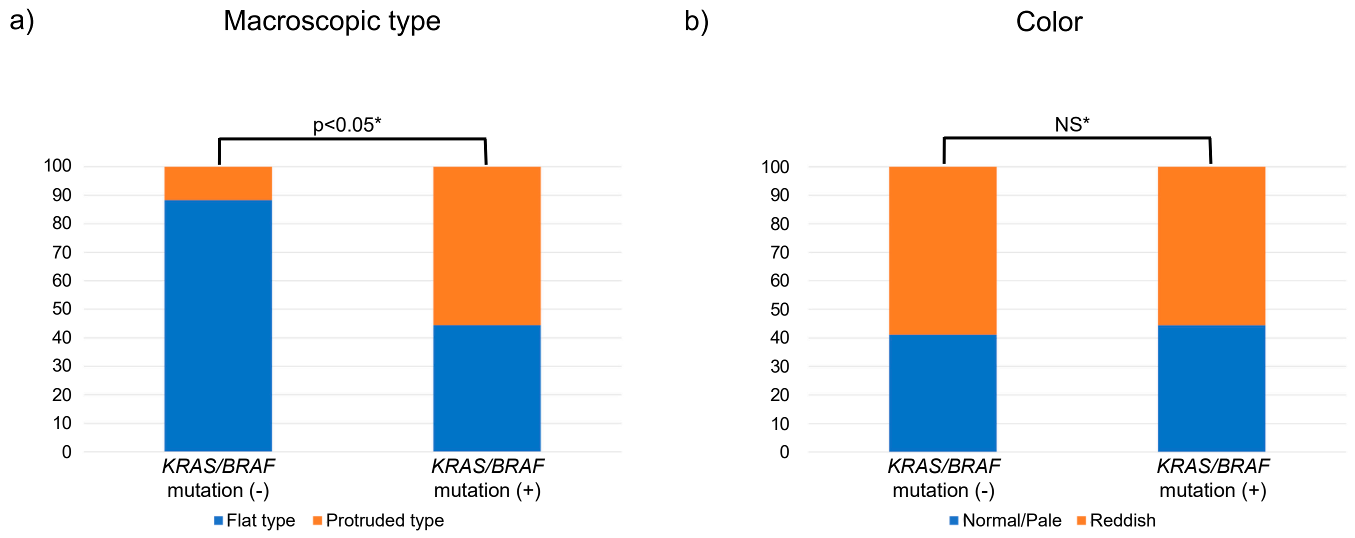

Figure S3: Correlation between *KRAS/BRAF* mutation status in biopsy specimens and endoscopic findings of the 40 early CRNUCs. The bar graphs show the correlation between *KRAS/BRAF* mutation in biopsy specimens and a) macroscopic type of tumor (flat type versus [vs.] protruded type), and b) color of tumor (normal/pale vs. reddish). CRNUC, colorectal neoplasia developing from ulcerative colitis mucosa; NS, not significant. *Categorical variables were compared using Fisher's exact tests.

Table S1: Details of the 40 genes included in the multi-gene panel in this study.

APC	*TCF7L2*	*NRG1*
TP53	*NWD1*	*MAP2K3*
KRAS	*ACVR1B*	*TPO*
PRKDC	*BRAF*	*TGFBR2*
CSMD3	*RNF43*	*MDM2*
CREBBP	*SMAD2*	*PIK3CA*
KIF26B	*ZNF536*	*SOX9*
LTBP4	*IL16*	
EP300	*IL17RD*	
ATM	*FBXW7*	

The multi-gene panel used in this study includes the same set of genes as the panel used in previous research.

Table S2: Clinicopathological features of colorectal neoplasia in step 1 (n = 14).

Variables	(%)
**Age (years old, median [range])**	57 [45–85]
**Sex**	
**Male**	5 (36)
**UC duration (years, median [range])**	19 [4–43]
**Type of UC**	
**Total colitis**	11 (79)
**Left-sided colitis**	3 (21)
**Clinical course of UC**	
**Relapse-remitting type**	5 (36)
**Chronic continuous type**	9 (64)
**Location**	
**Right-side colon**	2 (14)
**Left-side colon**	6 (43)
**Rectum**	6 (43)
**Background mucosa**	
**MES 0/1**	8 (57)
**MES 2/3**	6 (43)
**Resected method**	
**Endoscopic resection**	12 (86)
**Surgical resection**	2 (14)
**Histology**	
**High-grade dysplasia**	8 (57)
**Carcinoma**	6 (43)

UC: ulcerative colitis, MES: mayo endoscopic subscore

Table S3: Clinicopathological features of colorectal neoplasia in step 2 (n = 26).

Variables	(%)
**Age (years old, median [range])**	61 [38–78]
**Sex**	
**Male**	17 (65)
**UC duration (years, median [range])**	16 [0–36]
**Type of UC**	
**Total colitis**	17 (65)
**Left-sided colitis**	6 (23)
**Proctitis**	3 (12)
**Clinical course of UC**	
**Relapse-remitting type**	19 (73)
**Chronic continuous type**	6 (23)
**First attack type**	1 (4)
**Location**	
**Right-side colon**	7 (27)
**Left-side colon**	10 (38)
**Rectum**	9 (35)
**Background mucosa**	
**MES 0/1**	20 (77)
**MES 2/3**	6 (23)
**Resected method**	
**Endoscopic resection**	14 (54)
**Surgical resection**	12 (46)
**Histology**	
**Low-grade dysplasia**	6 (23)
**High-grade dysplasia**	9 (35)
**Carcinoma**	8 (30)
**Serrated lesion**	3 (12)

UC: ulcerative colitis, MES: mayo endoscopic subscore

Table S4: Correlation between the pathological diagnosis of resected specimens and the pathological diagnosis of biopsy specimens in 26 CRNUCs from step 2.

Pathological diagnosis of biopsy specimen [a]	Pathological diagnosis of resection specimen [b]		Total (%)
	UCAN (%)	Non-UCAN (%)	
**UCAN**	5 (100)	0 (0)	5 (100)
**Non-UCAN**	0 (0)	5 (100)	5 (100)
**Unclassified**	10 (63)	6 (37)	16 (100)

[a] The pathological diagnosis of biopsy specimens in this table refers to the pathological diagnosis by hematoxylin and eosin (HE) staining.

[b] The pathological diagnosis of resection specimens in this table refers to the final pathological diagnosis by HE staining with p53 and Ki67 immunostaining assessment.

CRNUC, colorectal neoplasia developing from ulcerative colitis mucosa; UCAN, ulcerative colitis-associated neoplasia.

Table S5: Correlation between the pathological diagnosis of resected specimens and p53 staining of biopsy specimens in the 26 CRNUCs in step 2.

p53 staining of biopsy specimen	Pathological diagnosis of resection specimen [a]		Total (%)	
	UCAN (%)	Non-UCAN (%)		
**Strongly positive**	8 (100)	0 (0)	8 (100)	$p<0.01$ [bc]
**Weakly positive**	4 (29)	10 (71)	14 (100)	
**Negative**	3 (75)	1 (25)	4 (100)	

[a] The pathological diagnosis of biopsy specimens and resection specimens in this table refers to the pathological diagnosis by hematoxylin and eosin staining with p53 and Ki67 immunostaining assessment.

[b] Categorical variables were compared using Fisher's exact tests.

[c] We compared a group with strongly positive p53 staining in biopsy specimens and a group with weakly positive/negative p53 staining.

CRNUC, colorectal neoplasia developing from ulcerative colitis mucosa; UCAN, ulcerative colitis-associated neoplasia.

Table S6: Correlation between the pathological diagnosis of resected specimens and the *TP53* mutation status of the biopsy specimens from the 26 CRNUCs in step 2.

TP53 **mutation**	**Pathological diagnosis of resection specimen** [a]		**Total (%)**	
	**UCAN (%)**	**Non-UCAN (%)**		
+	10 (91)	1 (9)	11 (100)	$p<0.01$ [b]
-	5 (33)	10 (67)	15 (100)	

[a] The pathological diagnosis of biopsy specimens and resection specimens in this table refers to the pathological diagnosis by hematoxylin and eosin staining with p53 and Ki67 immunostaining assessment.

[b] Categorical variables were compared using Fisher's exact tests.

CRNUC, colorectal neoplasia developing from ulcerative colitis mucosa; UCAN, ulcerative colitis-associated neoplasia.
